# Improving Hippocampal Memory Through the Experience of a Rich Minecraft Environment

**DOI:** 10.3389/fnbeh.2019.00057

**Published:** 2019-03-21

**Authors:** Gregory D. Clemenson, Caden M. Henningfield, Craig E. L. Stark

**Affiliations:** Department of Neurobiology and Behavior, University of California, Irvine, Irvine, CA, United States

**Keywords:** environmental enrichment, hippocampus, spatial exploration, video games, Minecraft

## Abstract

It is well known that the brain changes in response to the surrounding environment. The hippocampus has been shown to be particularly susceptible to environmental enrichment, with effects ranging from the generation of new hippocampal neurons and synapses to an increased expression of neurotrophic factors. While many of these changes in the hippocampus are well documented in animals, our understanding of how environmental enrichment can apply to humans is more ambiguous. In animals, spatial exploration has been shown to be a clear way to elicit the effects of environmental enrichment and considering the role of the hippocampus in spatial navigation, which has been shown in both animal models and humans, it suggests a viable avenue for translation of environmental enrichment to humans. Here, we test the hypothesis that the spatial exploration of a virtual video game environment, can impact the hippocampus and lead to an improvement in hippocampal-dependent memory. Using the video game Minecraft, we tested four groups of participants, each playing on custom servers and focusing on different aspects of Minecraft to test the effects of both building and exploration over the course of 2 weeks. We found an improvement in hippocampus-associated memory from pre-test to post-test and that the degree of improvement was tied to both the amount of exploration of the Minecraft world and the complexity of the structures built within Minecraft. Thus, the number of enrichment participants engaged in while playing Minecraft was directly correlated with improvements in hippocampal-dependent memory outside of the game.

## Introduction

The effects of environmental enrichment on the hippocampus have been well studied in animals. Simply exposing animals to an enriching experience that promotes sensory, physical, spatial, and cognitive stimulation can have an extremely positive impact on the hippocampus (Clemenson et al., 2018). While there is a vast amount of literature demonstrating the powerful influence of environmental enrichment in animals, how this manipulation applies to humans is less clear. Isolated factors such as exercise have been directly applied to humans with some success (Pereira et al., 2007; Erickson et al., 2009, 2010, 2011; Bugg and Head, 2011), but far less has been done in the area of spatial exploration. Studies in humans have shown that not only is the hippocampus active during spatial navigation like the rodent (Ekstrom et al., 2003; Jacobs et al., 2013), but structural changes can occur in the hippocampus with spatial training and expertise (Maguire et al., 1998, 2000; Woollett et al., 2009; Woollett and Maguire, 2011). Work in our lab and others has even shown that spatial training, in the form of playing immersive 3D video games, can alter hippocampal-dependent memory (Clemenson and Stark, 2015) and volume (West et al., 2017, 2018). Here, we show that focusing on specific aspects of the video game Minecraft can lead to improvements in hippocampal memory. Using custom servers to control the experience and record movement and interactions with the surrounding Minecraft environment, we found that this improvement is specifically correlated with the amount of spatial exploration and the complexity of structures built within the Minecraft world.

Exposing animals to an enriched environment can lead to increased hippocampal neurogenesis (Kempermann et al., 1997, 2002), synaptogenesis (Rampon et al., 2000; Gogolla et al., 2009), dendritic density and complexity (Moser et al., 1994; Faherty et al., 2003; Gonçalves et al., 2016), enhanced long-term potentiation (LTP; van Praag et al., 1999; Farmer et al., 2004), and increased expression of synaptic proteins and neurotrophic factors (Fabel et al., 2003; Nithianantharajah et al., 2004; Bekinschtein et al., 2011). Ultimately, these changes to the hippocampal circuitry lead to robust improvements in hippocampus-dependent cognition, including spatial learning and memory (Kempermann et al., 1997; Nilsson et al., 1999; van Praag et al., 1999, 2002; Sampedro-Piquero et al., 2013; Mora-Gallegos et al., 2015; Garthe et al., 2016) and pattern separation (Creer et al., 2010; Clemenson et al., 2015; Wu et al., 2015). Pattern separation is a computation designed to reduce interference between items in memory and thought to rely heavily on the dentate gyrus subfield of the hippocampus (McClelland et al., 1995; Yassa and Stark, 2011). Importantly, there is a clear and consistent positive relationship between hippocampal neurogenesis and pattern separation (França et al., 2017). In humans, we use the Mnemonic Similarity Task (MST; Kirwan and Stark, 2007; Stark et al., 2013) which was designed to index pattern separation by assessing behavioral responses to highly similar lure items after an initial study exposure. In addition, environmental enrichment has been shown to ameliorate many of the cognitive and functional deficits associated with rodent models of aging and other neurodegenerative disorders (Kempermann et al., 2002; Segovia et al., 2006; Leal-Galicia et al., 2008; Darmopil et al., 2009; Speisman et al., 2013).

In animal models, both exercise (van Praag et al., 1999; Kobilo et al., 2011; Mustroph et al., 2012; Vivar et al., 2013) and spatial exploration (Freund et al., 2013; Clemenson et al., 2015) have been shown to be core aspects of enrichment. For example, using radio frequency identification tags to track animal location, one study examined the exploratory behaviors of mice living in an enriched environment for 3 months (Freund et al., 2013). Mice exhibiting low spatial exploration tended to move back and forth between a few locations within the enrichment cage (e.g., between the nesting location and food location) whereas mice exhibiting greater spatial exploration tended to move unpredictably around the entire enrichment cage. Despite all mice were genetically identical, exploratory behaviors varied greatly amongst individual mice and further analysis revealed that individual exploratory behaviors positively correlated with hippocampal neurogenesis.

Given the relationship between the hippocampus in spatial navigation and memory in both humans and animals alike, we propose that the spatial exploration of a virtual video game environment is a human correlate of environmental enrichment and can lead to improvements in hippocampal function. Immersive 3D video game environments allow us to tease apart the contribution of spatial exploration with other elements of enrichment, such as exercise. Consistent with this hypothesis, we previously showed that playing the 3D video game Super Mario 3D World for 2 weeks could improve hippocampus-associated memory (Clemenson and Stark, 2015). Video games such as Super Mario 3D World often revolve around rich and immersive open-world environments for users to explore. Success in these games requires an awareness of the surrounding environment and a keen map knowledge to complete quests and find objectives. While the improvements we observed in hippocampus-associated memory correlated with video game performance there were several limitations of our study, many of which are common to most video game studies, that make it difficult to understand what about the experience led to the effect. First, our active control group played an entirely different game than our experimental group (Angry Birds vs. Super Mario 3D World). Although Angry Birds can still be considered an “engaging” game, it is inherently less spatial and therefore less complex and quite possibly less “enriching” than Super Mario 3D World. Second, the mechanics of each of these games are fundamentally different. Angry Birds is relatively simple and requires the user to launch a bird into a structure, while Super Mario 3D World requires several skills including problem-solving, jumping puzzles (specifically the timing of multiple jumps), and even interacting with enemies. Third, although our hypothesis revolves around the exploration of a virtual environment, it is difficult to quantify the spatial exploration of an individual while playing Super Mario 3D World as path data are not available. Collectible items within Super Mario 3D World are spread throughout the world and may provide a crude exploration metric, but these measures are magnitudes less sensitive than those used in animals (Freund et al., 2013).

While spatial exploration has clear relevance to enrichment and the hippocampus, the building aspect of Minecraft is more ambiguous. Despite no direct correlate of building in enrichment studies of humans or animals, there are several aspects of building that may be relevant to enrichment. First, enriched environments provide a certain amount of novelty to animals and although this has not been explicitly tested within the context of an enriched environment, we do know that the hippocampus plays a role in novelty recognition (Cohen and Stackman, 2015) and is greatly responsive to the exposure of a novel environment (Vazdarjanova and Guzowski, 2004; Tashiro et al., 2007). In building new structures, participants are constantly exposing themselves to this novelty and giving themselves rich episodes for the hippocampus to encode. In addition, they are learning a new cognitive skill of how to build potentially very complex structures. Thus, there is a great deal of non-spatial episodic information present in this condition. We should note, however, that while not explicitly spatial, the active process of building inherently becomes more spatial as building progresses. As the hippocampus plays a role in spatial mental imagery, including the ability to perceive objects or scenes from different perspectives (Hassabis et al., 2007; Bird et al., 2010; Summerfield et al., 2010) which may depend on the complexity of the scene or objects (Kim et al., 2013; Rungratsameetaweemana and Squire, 2018; Urgolites et al., 2018), the building condition should not be considered purely “non-spatial.” But, it has many rich non-spatial components and the scale of spatial involvement is certainly different than open-world exploration, giving us a different means within the same gaming environment of assessing whether enrichment, broadly defined, might have an impact on memory.

Here, we used the video game Minecraft and show that the spatial exploration of a virtual environment can improve hippocampus-associated memory. Minecraft is a sandbox game, which means that users are not restricted by game rules about the order of play. There is no right or wrong or winning or losing. It is a simple, endless, open world filled with mountains, deserts, forests, and oceans, for users to explore, create, and make their own. While all of our groups played Minecraft, they each focused on different combinations of the two main aspects of Minecraft: building and exploration. By creating completely custom Minecraft servers for every individual, we altered the focus of each group to promote building, exploration, or both. In addition, by being able to control their experience, we could manipulate the degree of sustained engagement in learning new aspects related to their activity (i.e., learning new skills or expanding their exploration of the world). Custom Minecraft servers also granted us the ability and control to track all movement as well as record the structures built in each world. Using the exact same metric of spatial exploration, our results parallel those in animals (Freund et al., 2013) and are consistent with our hypothesis that spatial exploration can improve hippocampus-associated memory. In addition, we found that the building of complex structures also influenced hippocampus-associated memory, suggesting that alternate novel, learning experiences found within Minecraft can be beneficial.

## Materials and Methods

### Participants

In total, 82 individuals (53 female, 29 male; mean age = 19.83, SD = 1.08) participated in the study. Participants played Minecraft at the UCI Esports Arena for 2 weeks (Monday-Friday), for a maximum of 45 min/day (7.5 h), however the actual time spent in the video game was slightly less (mean: 7.02 h, SD = 0.75) due to training, daily setup, and irregularities in participant schedules. All participants were recruited through advertisements posted around the UC Irvine campus and received compensation of $60, paid at the completion of the study. The advertisement specifically asked for participants with little to no experience with the video game Minecraft and did not reveal any details about the design or intention of the study, only that participants would play Minecraft for 2 weeks. All participants were screened for general video game experience, with an abbreviated version of the video game questionnaire used previously (Clemenson and Stark, 2015) including questions aimed at addressing both prior and current video game experience. We specifically screened against participants with prior Minecraft experience and knowledge. While many participants had prior experience with video games at one point in time, none of them actively played (<1 h/week) at the time of participation (within 3 months). This study was carried out in accordance with the recommendations of the Institutional Review Board (IRB) of the University of California at Irvine with written informed consent from all subjects. All subjects gave written informed consent in accordance with the Declaration of Helsinki. The protocol was approved by the Institutional Review Board (IRB) of the University of California at Irvine.

### UC Irvine Esports Arena

All Minecraft training took place at the UC Irvine Esports Arena located on the UC Irvine campus. The Esports Arena is home to 80 + high-end PCs, which were used by the participants to play Minecraft. Once the participants were trained on Minecraft, they were free to make their own hours and able to visit the arena anytime in the morning, between 9 am and 12 pm (when the Esports Arena was closed to the public), to continue their training. An experimenter was present at all times during training, to monitor progress and answer questions.

### Minecraft

Minecraft is a popular sandbox, or open-world, style video game that is practically infinite and is procedurally generated as the player explores. The entire Minecraft world is made up of different biomes (plains, jungles, deserts, forests, mountains, oceans, etc…) that are composed of different types of blocks, all of which can be manipulated by the player. There are no specific goals to the game and no right or wrong ways to play it. Minecraft is simply intended to be a video game world that players can creatively make their own.

All Minecraft participants played on individual custom servers, which were hosted on a local server in our lab. Custom servers were created using SpigotMC^1^ and a custom mod, Tracker (created by GDC), was added to track spatial locations of individuals and information about the world they created (e.g., blocks placed, current biome, time spent underground, etc.). All Minecraft participants within each group played on the exact same map but individually. The starting location was the same for every individual, ensuring that all participants received the same starting experience. All servers were set to “peaceful” which removed enemies and kept players health at full. Although difficult, it was still possible for players to die if they decided to swim in lava or fall off a mountain. Upon player death, they respawned at the initial starting point.

**Figure 1:**
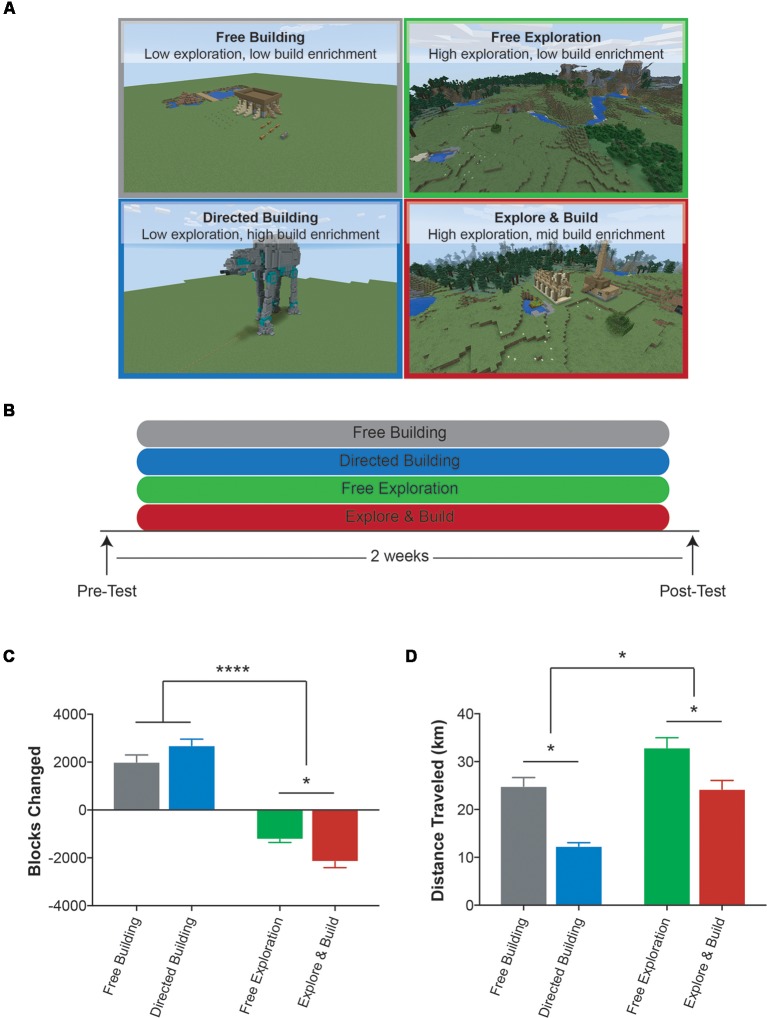
Description of the Minecraft groups and experimental design. **(A)** Example images of the four Minecraft groups used in this study: Free Building (gray), Directed Building (blue), Free Exploration (green) and Explore and Build (red). **(B)** Experimental design. **(C)** The average total number of blocks changed for each Minecraft group over the course of the study. A positive number indicates an overall addition of blocks to the world and a negative number indicates an overall subtraction of blocks from the world. **(D)** The average total distance (km) traveled by each Minecraft group over the course of the study. All data are presented as mean ± SEM, *p < 0.05, ****p < 0.0001.

Participants were randomly assigned to one of four Minecraft groups in a 2 × 2 design focusing on building vs. exploring activities (to vary the type of enrichment) and undirected vs. directed activities (to vary the degree of enrichment): Free Building (11 females, 10 males; mean age = 20.4, SD = 1.27; low exploration, low building enrichment), Directed Building (13 females, six males; mean age = 19.4, SD = 0.61; low exploration, high building enrichment), Free Exploration (16 females, seven males; mean age = 19.88, SD = 1.22; high exploration, no building enrichment), and Explore and Build (13 females, six males; mean age = 19.58, SD = 0.84; high exploration, mid building enrichment; Figures 1A,B). While participants knew that they were going to play Minecraft, they were not told any details about the study and were blind to the group they were in. To prevent participants from observing other individuals from other groups, each Minecraft group was split into two cohorts and each cohort played in the UCI Esports Arena during the same 2 weeks. A Minecraft tutorial was given to all study participants, with instructions on how to build a house, which ultimately became their “home.” While all Minecraft groups were given the same instructions for PC controls and taught the basics of Minecraft, each group was told to focus on a different aspect of Minecraft: building or exploring. In addition, they differed in how goal-oriented these aspects were and, as a result, the degree of engagement in pursuing new challenges in the area.

The Free Building and Directed Building groups were asked to focus on the building aspect of Minecraft. Both groups played on a completely flat world (no biomes, no mountains, no lakes, no caves, etc.) with no animal or vegetation spawns. While both groups focused on building, the main difference between the Free Building and the Directed Building groups was that the Directed Building group was given challenges to continuously expose them to new aspects of building and thereby provide a potentially higher level of enrichment. We did so by directing them to build complex structures found on grabcraft^2^ such as an AT-AT Walker (Figure 1A lower left box, 3,785 blocks) and Spongebob’s House (3,486 blocks). Once a structure was chosen, the necessary blocks to build the structure were placed into a chest in the world. During the first week, participants in the Directed Building group were limited to pre-selected structures to ensure they had a grasp on the controls and mechanics of Minecraft. During the second week, they were allowed free choice of structure. In contrast, the Free Building group was given chests full of blocks but were not given any specific direction as to what they could or should build. During the second week, to maintain engagement, participants in the Free Building group were given new items to build with redstone—Minecraft’s tool for powering items and creating circuits.

The Free Exploration group played Minecraft in the most traditional sense. All participants played on individual servers, using the exact same map [seed: 1932176428914146755, starting point (x, y, z): −312, 73, −420] with easy access to resources. After creating their “home” at the starting spawn location, participants were told they could play Minecraft however they wished. The only rule was that at the end of their training session, they had to end up at their “home.” This encouraged participants to learn about the surrounding area and preventing anyone from walking in a straight line endlessly for the duration of the study. Thus, they were allowed to engage in as much or as little spatial exploration as they wished and therefore have as much or as little potential enrichment as they wished. To maintain engagement, at the beginning of the second week, participants were given a scavenger hunt list of objects and places to find to encourage further exploration of the Minecraft world. This scavenger hunt list included flowers, gems, resources, waterfalls, villages, and other objects/locations that were in the local areas.

The Explore and Build group was designed to bring in both potential avenues of enrichment by combining the two activities. The first week was the same as the first week of the Free Exploration in which they explored and learned the spatial layout of their area. The second week was modeled on the Directed Building, but rather than providing them with free access to all needed materials for their structure of choice, participants had to find these materials in the world themselves. Therefore, this pushed heavily on both learning complex construction techniques and on spatial exploration as they searched for the needed materials.

### Mnemonic Similarity Task (MST)

**Figure 2:**
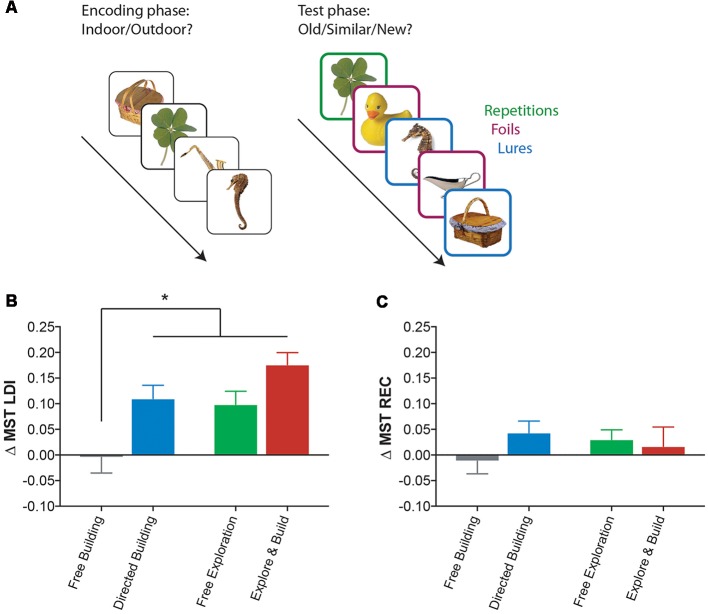
Playing Minecraft leads to improvement on a hippocampal memory task. **(A)** A schematic of the Mnemonic Similarity Task (MST), an object recognition memory task that uses not only traditional repeated and unstudied foil items, but also highly similar lures and a three-choice response. **(B)** Change in the hippocampal-mediated lure discrimination index (LDI) performance (Pre-Test to Post-Test) of the MST, for all Minecraft groups. **(C)** Change in general recognition (REC) performance (Pre-Test to Post-Test) of the MST, for all Minecraft groups. All data are presented as mean ± SEM, *p < 0.05.

One of our primary outcome variables was the lure discrimination index (LDI) of the MST (Kirwan and Stark, 2007; Stark et al., 2013), which was designed to behaviorally index pattern separation and hippocampal function. The MST is now a widely used behavioral task and has been repeatedly shown to be sensitive to age-related memory decline and to hippocampal connectivity (Yassa et al., 2010a, 2011b; Bennett and Stark, 2015; Bennett et al., 2015) and function (Bakker et al., 2008, 2012, 2015; Yassa et al., 2010b, 2011a,b) and even to the alteration of hippocampal function following treatment for MCI with Levetiracetam (Bakker et al., 2012, 2015). There are two phases of the MST (Figure 2A). The first phase is an implicit encoding phase in which participants are shown pictures of everyday objects and asked to make a simple “indoor/outdoor” judgment. The second phase is an explicit test phase in which participants are shown everyday objects that are either the same (target), similar (lure), or different (foil) to the items encoded in the first phase. Participants then make “old/similar/new” judgments for each of these objects. Two metrics are used during the analysis. The LDI is the metric that has been shown to be sensitive to DG/CA3 activity and is defined as the probability of correctly identifying a lure item as “similar,” correcting for any response bias using the probability of incorrectly identifying a foil item as “similar”: p (similar|lure) − p (similar|foil). The recognition index (REC) is a standard object recognition memory score, defined as the probability of correctly identifying a target item as “old” minus the probability of incorrectly identifying a foil item as “old”: p (old|target) − p (old|foil).

### Roaming Entropy (RE)

Roaming entropy (RE) is a measure of spatial exploration that was previously used to measure the exploratory behavior of mice living in an enriched environment (Freund et al., 2013). In the equation below, *p*_i,j_ is the probability of participant *i* being at location *j*. The RE is defined as the Shannon entropy of the roaming distribution of participant *i*, where *k* is the total number of possible locations. Dividing by log (k) scales the range from 0 to 1.

$${\text{RE}}_{i}=\sum_{j=1}^{k}\left(p_{i,j}\log(p_{i,j}\right))/\log(k)$$

In the mouse study (Freund et al., 2013), mice were tagged with radio frequency ID tags and were monitored by antennas placed around the enriched environment. Essentially, RE is a measure of how likely a mouse would be located at each target location (antenna), at any given time. A lower RE value reflects that animal’s movements were more predictable, and therefore exhibited less exploration, and a higher RE value reflects that the animal’s movements were less predictable, and therefore exhibited more exploration. In our study, we used the same RE measure to assess the spatial exploration of participants as they explored Minecraft. A virtual location was placed every 10 meters in the x-, y-, and z- axes (mirroring the antennae locations). We calculated the probability that an individual would be at each location at any given time, summed across all locations in the world that could have been visited in the allotted time. A lower RE value indicates that a participant’s position was more predictable (lower spatial exploration) whereas a higher RE value means a participant’s position was less predictable (higher spatial exploration).

### Principal Components Analysis (PCA)

We performed a principal components analysis (PCA; MATLAB_R2018a) on the spatial location data collected from each participant as he or she moved through the world to determine the amount of variance each variable (x-, y-, and z-) contributed to the overall variance. Participant’s total coordinate data was split into 60-s chunks and each 60-s dataset was entered into a PCA, providing a vector describing the relationship of the three variables (x-, y-, and z-). Using the coefficients for each variable (individual values that make up the normalized vector of each principal component), we first averaged within the subject (across 60 s blocks) and then across all subjects in a group. We then calculated the percent variance contributed by the y variable $\left(\frac{y}{x+y+z}\right)$.

### Statistical Analyses

Statistical analyses were conducted using Prism 7 (GraphPad Prism). Planned comparisons between groups were performed using two-way analysis of variances (ANOVAs), without repeated measures, and Sidak correction for multiple comparisons. General linear regression models (GLMs) were used to analyze correlations between group metrics. Shapiro-Wilk normality test revealed that distributions did not significantly deviate from the normal distribution. A significance value of 0.05 was used for all statistical analyses.

## Results

### All Four Groups Focused on Different Aspects of Minecraft

Participants were randomly assigned to one of four groups that varied in both the central activity (exploring vs. building) and the amount of engagement in that activity (Figures 1A,B). The two groups that focused on building, Free Building and Directed Building, were placed in a flat and featureless world and simply asked to build. While the Free Building group was given limitless resources and little guidance, the Directed Building group was instructed on how to build progressively more complex structures. The Free Exploration and Explore and Build groups both played on rich, computer-generated worlds typical of Minecraft and were simply asked to explore the world. The Explore and Build group was a combination of building and exploring. While the first week was the same as the Free Exploration group, during the second week of training, they were required to scavenge for resources needed to build a complex structure.

In analyzing their behavior within the game, as expected, the four groups differed in the most basic measures of building and exploration. The Free Building and Directed Building groups focused on building far more than exploring, while the Free Exploration and Explore and Build groups focused heavily on exploring, with the Explore and Build partaking in both (Figures 1C,D). For each participant, we used the number of blocks changed in the world as a measure of the amount of building (Figure 1C; a positive value is a cumulative addition of blocks to the world and a negative value is a cumulative subtraction of blocks from the world) and analyzed the scores using a 2 × 2 ANOVA with factors for the type of activity (building vs. exploring) and whether the activity was directed or not (free vs. directed). We found a significant main effect of type of activity (two-way ANOVA: *F*_(1,78)_ = 285.8, *p* < 0.0001) with building groups placing more blocks in the environment and the active exploring groups removing more blocks from the world (removing blocks is often necessary to explore and find resources in the world) and a significant interaction (two-way ANOVA: *F*_(1,78)_ = 11.44, *p* < 0.01). A *post hoc* analysis (Sidak correction for multiple comparisons at *p* < 0.05) revealed that while there was no difference between building groups (Free Building vs. Directed Building), the Explore and Build removed significantly more blocks from the world when compared to the Free Exploration group, consistent with their need to harvest the necessary resources to build a structure during the second week.

As an initial measure of the amount of exploration, we calculated the total distance that individuals walked while playing Minecraft (Figure 1D). Here, we found a significant main effect of activity type with the exploring groups walking more than the building groups (two-way ANOVA: *F*_(1,78)_ = 31.05, *p* < 0.0001) and a main effect of amount of engagement with the free groups walking more than the directed groups (two-way ANOVA: *F*_(1,78)_ = 35.07, *p* < 0.0001). A *post hoc* analysis (Sidak correction for multiple comparisons at *p* < 0.05) revealed that the Free Building and Explore and Build groups were similar, but all other comparisons differed reliably. It is interesting to note that in terms of distance, the Free Building group traveled more than the Directed Building group and a similar amount to the Explore and Build group. One source of this is that the Free Building group had to continually return to the chest in order to retrieve resources to build. Unlike the Directed Building group, which had a specific plan for building, the building process of the Free Building group was unconstrained. Without a specific goal in mind, this free-form building group was apparently far less efficient in their trips to and from the supply chest. Regardless, when combined with the data above on building, it is clear that all four Minecraft groups were different in how they interacted with the environment.

### All but the Free Building Group Showed Improvements in Hippocampus- Associated Memory

Our initial question was whether playing Minecraft, focusing on either building, exploring or both, can improve hippocampus associated memory. Using the change in the MST’s LDI (post-test minus pre-test, Figure 2B) as a measure of change in hippocampal function, we found a significant main effect of activity type (two-way ANOVA: *F*_(1,78)_ = 10, *p* < 0.01) and amount of engagement (two-way ANOVA: *F*_(1,78)_ = 12.89, *p* < 0.001). A *post hoc* analysis (Sidak correction for multiple comparisons at *p* < 0.05) revealed that not only were the Directed Building, Free Exploration, and Explore and Build groups all showing reliable changes in the LDI score, but all three groups showed reliably greater improvements than the Free Building group (which did not differ from zero, one-sample *t*-test *t*_(20)_ = 0.15, *p* = 0.87). While there was some evidence for an additive effect in the Explore and Build condition relative to the Directed Building (*p* = 0.06, uncorrected) and Free Exploration (*p* = 0.03, uncorrected), these did not survive corrections for multiple comparisons. Importantly, there was no change in general recognition memory measure in the MST (Figure 2C), indicating a specific improvement in the highly hippocampal-dependent lure discrimination metric.

### LDI Improvement Correlated With Spatial Exploration of the Minecraft World but Not Total Distance Traveled

**Figure 3:**
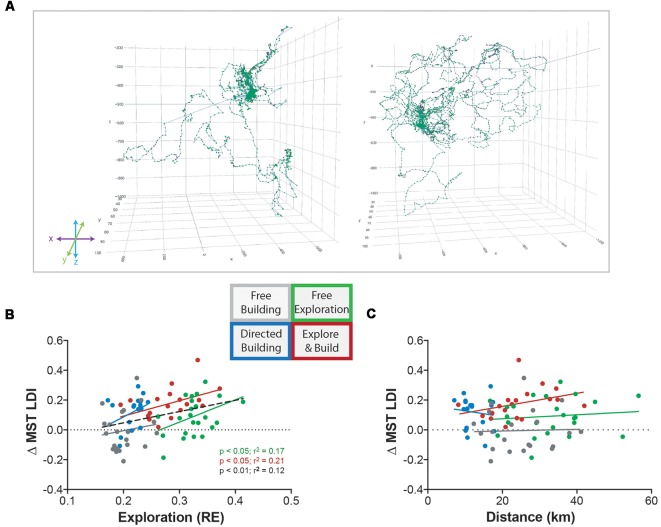
Exploration of Minecraft correlates with improvement in hippocampal memory. **(A)** Example path traces of low exploration (left) and high exploration (right) individuals from the Free Exploration group. **(B)** The relationship between the change in MST LDI performance (Pre-Test to Post-Test) and the amount of exploration roaming entropy (RE). Colors correspond to the Minecraft group indicated by the diagram: Free Building (gray), Directed Building (blue), Free Exploration (green), and Explore and Build (red). The black dotted line represents the combination of all four Minecraft groups together. Significant statistics are denoted by group color including black for all groups. **(C)** The relationship between the change in MST LDI performance (Pre-Test to Post-Test) and the total distance traveled (km). Colors correspond to the groups as previously described.

One of our main hypotheses was that environmental enrichment via the exploration of the Minecraft world would be important for the observed improvement in hippocampal behavior. Similar to spatial exploration found in mice (Freund et al., 2013) we found that our human participants varied greatly in their exploratory behavior (Figure 3A). Even within a group, some explored the world far more than others. As in the rodent work, we used RE to measure the amount of exploration (see “Materials and Methods” section). Participants with lower exploration RE scores (e.g., Figure 3A, left) tended to focus their exploration around the spawn area (where their house was built), whereas participants with higher exploration RE scores (e.g., Figure 3A, right) were more likely to adventure further in all directions. As expected, we found a main effect of activity type with the exploring groups (Free Exploration and Explore and Build) showing higher RE values than the building groups (two-way ANOVA: *F*_(1,78)_ = 170.2, *p* < 0.0001; data not shown).

More important than this simple group effect, however, is the relationship between the amount an individual explored the world and their subsequent memory improvement. When examined individually, we found a significant correlation between RE and ΔLDI in both exploring groups (Free Exploration: *R*^2^ = 0.17, *p* < 0.05; Explore and Build: *R*^2^ = 0.21, *p* < 0.05; Figure 3B), with effects in the two building groups not being reliable on their own. Importantly, in no group was the total amount of distance covered correlated with ΔLDI (Figure 3C), indicating that the quality of exploration was important for the improvement in LDI. Further supporting this conclusion, when analyzing the relationship between RE and ΔLDI across all groups, there was not only a reliable correlation (*R*^2^ = 0.012, *p* < 0.01), but when testing whether a single slope fits all datasets or whether the slope differed, we found no evidence for the use of differing slopes (Extra sum of squares *F*_(4,154)_, *p* = 0.8) suggesting a common relationship between the amount of exploration and improvement in memory regardless of condition.

### LDI Improvement Correlated With Active Building More Complex Structures

The Directed Building demonstrated a clear and significant improvement in LDI performance when compared to Free Building, who also focused on building. We wanted to better understand the structures that both Directed Building and Free Building created. One clear difference between these two groups was that the structures the Directed Building created were denser and seemingly more complex, with the Directed Building adding more blocks to the environment (Figure 1C) and walking less (Figure 1D) than all other groups. Assessing “complexity” of their building activity is not as straightforward as measuring something like RE as there is no established metric of building activity or complexity. Furthermore, building is an active process with a constant exchange between the placement and removal of blocks, a factor that we cannot reasonably address with our current dataset. What we would like to capture is whether their building is a simple structure or something more challenging that required more planning to physically construct (e.g., building stairs or scaffolds to reach locations during construction). Here, we use three different measures to address building activity in Minecraft.

**Figure 4:**
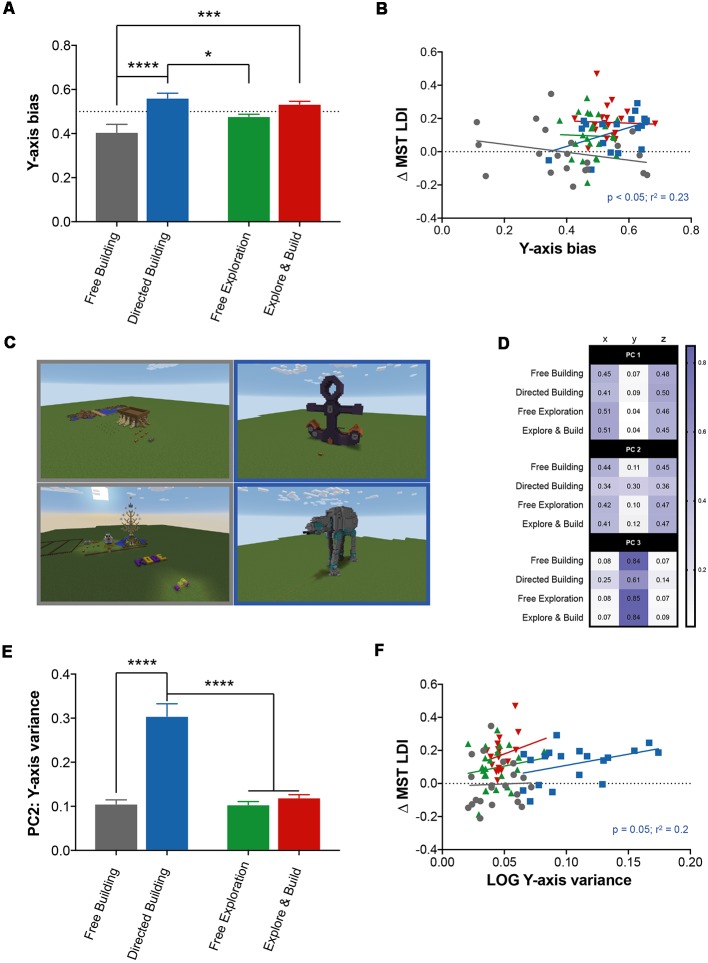
Building in Minecraft correlates with improvement in hippocampal memory. **(A)** The y-axis bias score represents the amount of spatial exploration (RE) along the vertical axis (y axis) compared to the horizontal axes (x- and z-axes) for all Minecraft groups. The dotted line represents a value where exploration along the vertical axis is equal to exploration along the horizontal axes. **(B)** The relationship between the change in MST LDI performance (Pre-Test to Post-Test) and the y-axis bias score. Minecraft groups are represented by their corresponding colors with significant statistics denoted by group color. **(C)** Example images from the Free Building (left column) and Directed Building (right column) groups. **(D)** Overview of the principal components analysis (PCA) for all Minecraft groups. Each value represents the amount of variance explained by the variable (column) for each Minecraft group (row), for all three principal components (PC1, PC2, and PC3). **(E)** The amount of variance explained by the y variable of all Minecraft groups for the second principal component. **(F)** The relationship between the change in MST LDI performance (Pre-Test to Post-Test) and the LOG of the y-axis variance of the second principal component. All data are presented as mean ± SEM, *p < 0.05, ***p < 0.001, ****p < 0.0001.

Perhaps the simplest metric that could capture this complexity is the block-change density, or the number of blocks changed per square kilometer. Using this metric, we found a significant main effect of activity type (two-way ANOVA: *F*_(1,78)_ = 259.4, *p* < 0.0001; data not shown), amount of engagement (*F*_(1,78)_ = 13.13, *p* = 0.0005), and an interaction (*F*_(1,78)_ = 53.2, *p* < 0.0001). A *post hoc* analysis (Sidak correction for multiple comparisons at *p* < 0.05) revealed that the Directed Building group had a higher block-change density than all other groups and that the Free Building group had a higher block-change density than the two exploring groups. As building complex structures in Minecraft (e.g., Figure 4C) typically requires significant movement vertically (to place blocks more than one unit above you, you must build some form of stairs to reach that level), block-change density is only a very rough, indirect measure of this complexity. We created two metrics to assess the degree of variance along the vertical (y) axis.

First, using the previous measure of spatial exploration (RE), we calculated a RE value for each individual axis, which allowed us to individually quantify the predictability of movement along each axis. We predicted that if Directed Building were in fact building taller structures, they would spend more time exploring the vertical axis (i.e., greater unpredictability) compared to the other groups. Using this measure, we created an index score to measure the amount of vertical-axis bias $\left(\frac{yRE}{xRE+zRE}\right)$ for all individuals (Figure 4A). We found no evidence for an effect of activity type, but a significant main effect of amount of engagement (two-way ANOVA: *F*_(1,78)_ = 22.92, *p* < 0.0001) and a significant interaction between activity type and amount of direction (two-way ANOVA: *F*_(1,78)_ = 4.976, *p* < 0.05) with the directed groups (Directed Building and Explore and Build) scoring significantly higher on this index compared to both exploring groups (Free Building and Free Exploration). A *post hoc* analysis (Sidak correction for multiple comparisons at *p* < 0.05) revealed Directed Building scoring reliably higher than both Free Building and Free Exploration whereas Explore and Build only scored reliably higher than Free Building. Furthermore, a positive correlation was found in Directed Building between the y-axis bias index score and ΔLDI in Directed Building (*R*^2^ = 0.22, *p* < 0.05) but not in other groups (Figure 4B).

Second, we used PCA to measure how much of the variability of movement was contributed by the movement along the vertical axis. Each coordinate data set was split into 1-min blocks and PCA was used to characterize the variance in location within that time and determine how much the total motion was driven by motion along each axis (x, y, and z). The resulting coefficients from the three PCs were first averaged within the subject (across 1-min blocks) and then across all subjects in a group. The first principal component (PC1) accounted for 83%–89% (across groups) of the variance in motion and was heavily dominated by movement along the horizontal plane (x and z axes; Figure 4D). Despite the high degree of similarity, we found a significant main effect of activity type (two-way ANOVA: *F*_(1,78)_ = 10.85, *p* < 0.01; data not shown) with the building groups (Free Building and Directed Building) having a higher contribution of y-axis movement (~8%) to PC1 than the exploring groups (Free Exploration and Explore and Build, ~4%). The second PC (PC2) accounted for 10%–15% of the variance and showed main effects of both activity type (two-way ANOVA: *F*_(1,78)_ = 45.45, *p* < 0.0001) and amount of engagement (two-way ANOVA: *F*_(1,78)_ = 61.85, *p* < 0.0001) along with a significant interaction between the two (two-way ANOVA: *F*_(1,78)_ = 43.72, *p* < 0.0001; Figure 4E). Much of the observed effects can be attributed to the Directed Building as a *post hoc* analysis (Sidak correction for multiple comparisons at *p* < 0.05) revealed a highly significant difference between the Directed Building and all other groups. Their movement along the y-axis contributed to ~30% of the total variance in PC2 compared to ~11% for all other groups. There was a clear and positive correlation between y-axis variance of Directed Building and ΔLDI (*R*^2^ = 0.2, *p* = 0.05; Figure 4F). Lastly, although PC3 only accounted for ~1% of the total variance, the y-axis variable contributed to significantly more of the variance in the Free Building, Free Exploration and Explore and Build groups than the Directed Building (Multiple comparisons: *p* < 0.0001 for all groups; data not shown; Figure 4D). These data indicate that the y-axis variable plays a bigger role in the Directed Building than in all other groups.

## Discussion

We set out to replicate and improve upon our prior report that video games may act as human analogs for environmental enrichment (Clemenson and Stark, 2015) and to explore the effects of the kind of enrichment and the amount of enrichment had on memory ability. Using the video game Minecraft, we constrained the environment to encourage participants to focus on either spatial exploration or building and we promoted greater levels of engagement in new, more challenging forms of each activity by use of directed, or guided activities within the game. We again observed improvements in a hippocampal-dependent memory task, unrelated to the video game activity, following 2 weeks of gameplay (Figure 2B) with results indicating that both the kind of enrichment activity and the degree of engagement in the activity were correlated with the amount of improvement.

Even without directed activities to enhance engagement, we found that the spatial exploration of a Minecraft environment led to greater improvements in hippocampal cognition. In animals, spatial exploration of an enriched environment has been tied to hippocampal neuroplasticity (Freund et al., 2013). Using Minecraft as our enriched environment, we translated this animal study to humans and found similar results. Using the exact same methodology and equation for exploration (RE), we found that higher spatial exploration, but not overall distance walked, led to greater improvements on a hippocampal memory task. As expected, the two groups that focused on the exploring aspect of Minecraft (Free Exploration and Explore and Build) scored the highest on our exploration metric. However, it is important to note that when all four groups were combined (Figure 3B), we observed a highly significant correlation with hippocampal memory which suggests an important notion about building in Minecraft. Although Free Building and Directed Building were on the lower end of the exploration metric, participants were still able to explore an environment regardless of how simple it might be. While we tried to limit spatial exploration of both Free Building and Directed Building, their environments became progressively more spatial as they began to build structures and added to the uniqueness of their own individual worlds. With more time, Free Building and Directed Building might naturally increase their exploratory behaviors as they build and make their environment more spatial. In addition, this correlation highlights the fact that, similar to the animal studies, spatial exploration is a powerful factor driving our observed results and that its effects may be independent of other factors underlying enrichment’s effects.

In addition to spatial exploration, our results showed that building within Minecraft can also lead to improvements in hippocampal-based memory. The effects were less robust as simply giving people the world to explore (Free Exploration) led to reliable effects, while simply giving people the resources needed to build structures (Free Building) was insufficient. For the building to be effective at improving memory, participants needed to be directed towards building activities that had them build new, more complex structures (Directed Building). While the Directed Building consistently placed higher in all three metrics of building activity, results from the other groups (Free Building, Free Exploration and Explore and Build) were mixed. Since building is an active process, it is possible that we are only capturing a portion of building complexity. Further analyses are necessary to better understand the contribution of the building process. The environmental enrichment literature strongly implicates spatial exploration as a powerful means to induce the effects on the hippocampus (as observed here as well), but the translation of the building aspect of Minecraft is less clear. In part, this may be because there simply are not analogous experiments in the rodent. However, several explanations recapitulate a common question in the hippocampal literature: whether the observed results stem from spatial aspects of the Directed Building group, such as spatial mental imagery, or whether they are the result of non-spatial forms of novelty and learning, building in Minecraft allows users to create their own unique, complex and spatial environments. As mentioned previously, building is a way to change the surrounding environment and inherently makes an environment more spatial. After all, cities are a product of building within our own environments and the complex structures and the spatial nature of modern cities require maps to navigate. In fact, many studies in humans specifically use city environments to understand the role of the hippocampus in navigation. However, building new items and structures in Minecraft, and learning how to build them, is a novel learning experience for participants outside of anything spatial *per se*. While we cannot be resolute on this matter as the interpretations discussed here are merely speculation, it was the case that being pushed to learn to create new, complex structures (Directed Building) had as big an effect on memory improvement as free exploration of the world (Free Exploration) despite far lower amounts of exploration (RE), supporting the notion that at least some of the observed effect was likely non-spatial. In addition, the Explore and Build group showed roughly twice the improvement in memory that the Free Exploration group showed, despite having half of the exploration-driven gameplay directed towards learning to build new structures. Thus, we conclude that spatial exploration alone has a clear effect, but that learning new skills (directed groups) can have a similar effect, possibly non-spatial in nature.

Here, we show that playing the video game Minecraft can positively impact hippocampal function in the form of improved memory performance on tasks unrelated to activities in the game itself. Both spatial exploration and building were at least partly responsible for this observed improvement. Whether these aspects are able to improve performance on other hippocampus-related behaviors is still unknown, however, we did observe an improvement in a virtual water maze task in our previous study using video game enrichment. We would predict similar results given that the exploration of a virtual environment like Minecraft has more direct relevance to a virtual water maze than the MST task. While we previously demonstrated that 3D video games can have an influence on hippocampal function using the video game Super Mario 3D World (Clemenson and Stark, 2015), this study improves upon the previous study for several critical reasons. First and foremost, while Minecraft is not completely open-source, it is customizable which affords great flexibility and freedom to create environments on private servers where we can entice users to focus on distinct aspects of the game, while still being able to both track player movement and the addition/removal of blocks from the world. Second, this control over the environment and ability to track participants allowed us to implement the same metrics of spatial exploration as in rodent studies, giving us a better understanding of how environmental enrichment relates to humans. Lastly, all groups in this study played Minecraft. Several studies have explored the effects of video games on brain function, however, a major caveat of many of these studies is the fact that the controls and experimental groups play entirely different games. All video games differ on several levels, including game mechanics, game dynamics, visual and audio features, engagement, etc. These inherent differences add a level of uncertainty and can reduce the reliability of the interpretations. Often, this simply cannot be helped as commercial video games are stuck with a set of rules that make it difficult to deviate from. The video game Minecraft can address these limitations and sets itself apart as a platform for video game research.

It is well understood that the hippocampus is a region of great neuroplasticity and is constantly shaped by environmental experiences. Results from this study are consistent with animal research and support the idea that video games can act as a form of environmental enrichment in humans. While specific aspects of the enrichment manipulations used in animals are simply not present in video games, the virtual worlds found in modern video games provide a rich and complex environment for people to experience and offer a real opportunity for intervention in humans.

## Data Availability

The datasets generated for this study are available on request to the corresponding author.

## Author Contributions

GC and CS designed the experiment, analyzed the data and wrote the article. GC and CH collected the data.

## Conflict of Interest Statement

The authors declare that the research was conducted in the absence of any commercial or financial relationships that could be construed as a potential conflict of interest.

## Footnotes

^1^www.spigotmc.org

^2^www.grabcraft.com
